# Impact of the Neonatal Resuscitation Video Review program for neonatal staff: a qualitative analysis

**DOI:** 10.1038/s41390-024-03602-9

**Published:** 2024-10-04

**Authors:** Zoe Weimar, Debra Nestel, Alexis Battista, Samantha Best, Arunaz Kumar, Douglas A. Blank

**Affiliations:** 1 Monash University, Monash School of Medicine, Wellington Road, Clayton, VIC 3800 Australia; 2 The University of Melbourne, Department of Surgery, Melbourne, VIC 3010 Australia; 3 Monash University, School of Clinical Sciences, Clayton, VIC 3168 Australia; 4 Uniformed Services University of the Health Sciences, School of Medicine, 4301 Jones Bridge Rd, Bethesda, MD 20814 USA; 5 Monash Newborn, Monash Health, 246 Clayton Rd, Clayton, VIC 3168 Australia; 6 Monash University, Faculty of Medicine, Nursing and Health Sciences, Clayton, VIC 3168 Australia; 7 Monash University, Department of Paediatrics and The Ritchie Centre, Hudson Institute of Medical Research, Clayton, VIC 3168 Australia

## Abstract

**Background:**

Neonatal resuscitation video review (NRVR) involves recording and reviewing resuscitations for education and quality assurance. Though NRVR has been shown to improve teamwork and skill retention, it is not widely used. We evaluated clinicians’ experiences of NRVR to understand how NRVR impacts learning and can be improved.

**Methods:**

Neonatal Intensive Care Unit (NICU) clinicians with previous NRVR experience were recruited for individual semi-structured interviews. Using a social constructivist viewpoint, five researchers used thematic analysis to analyze participant responses.

**Results:**

Twenty-two clinicians (11 nurses, 11 doctors) were interviewed. All participants expressed positive attitudes towards NRVR. Four themes were identified: (1) Learning from reality—exposure to real-life resuscitations was highly clinically relevant. (2) Immersive self-regulation—watching videos aided recall and reflection. (3) Complexities in learner psychological safety—all participants acknowledged viewing NRVR videos could be confronting. Some expressed fear of judgment from colleagues, though the educational benefit of NRVR superseded this. (4) Accessing and learning from diverse vantage points—NRVR promoted group discussion, which prompted participant learning from colleagues’ viewpoints.

**Conclusion:**

Neonatal clinicians reported NRVR to be an effective and safe method for learning and refining skills required during neonatal resuscitation, such as situational awareness and communication.

**Impact:**

- Neonatal resuscitation video review is not known to be widely used in neonatal resuscitation teaching, and published research in this area is limited.
- Our study examined clinician attitudes towards an established neonatal resuscitation video review program.
- We found strong support for teaching using neonatal resuscitation video review among neonatal doctors and nurses, with key benefits including increased situational awareness and increased clinical exposure to resuscitations, while maintaining psychological safety for participants.
- The results of this study add evidence to support the addition of video review to neonatal resuscitation training.

## Introduction

Neonatal resuscitation is a medical emergency in which clinicians facilitate a newborn’s transition from fetal to extrauterine life, following a set of guidelines suggesting key interventions.^1^ Approximately 5% of babies born at term and over 85% of babies born very preterm (less than 32 weeks gestation) will require resuscitative interventions at birth.^2,3^ Clinicians must rapidly assess the newborn’s condition, analyze physiological data, and communicate effectively in often chaotic environments, with this complexity sometimes resulting in errors and guideline deviations.^4,5^

As skill retention has been shown to decrease following training, ongoing education is essential to prevent deterioration in resuscitation skills and thereby reduce error rates.^6–8^ While post-simulation debriefings (e.g., an intentional and often guided discussion following a simulation encounter to promote reflection) have been shown to facilitate learning after simulated resuscitations, opportunities to debrief after real resuscitations are often limited and affected by recall bias.^9–11^

Neonatal resuscitation video review (NRVR) describes an educational technique wherein resuscitations are recorded, viewed, and discussed with neonatal clinicians. In some cases, clinicians view their performances, but in others, they view purposively selected resuscitations that they may not have participated in. This process enables comprehensive reflection and exposes clinicians to a broader range of clinical situations.^12,13^ Implementation of similar programs has been shown to improve clinician performance in trauma resuscitation and laparoscopic surgery.^14,15^ Furthermore, NRVR offers clinicians access to resuscitations managed by colleagues, thus overcoming limitations presented by rostering.

Published evidence evaluating the impact of NRVR is primarily positive, with NRVR shown to be associated with improved teamwork in resuscitation, improved compliance to resuscitation algorithms, increased understanding of the physiological changes during neonatal resuscitation, and improved skill retention.^12,16–21^ Video review has also recently been shown to be effective in identifying areas for improvement in neonatal procedures—guiding protocol changes and development of educational programs.^22^

Previous studies evaluating clinicians’ attitudes towards video review (VR) in obstetric emergencies and trauma resuscitations have found that clinicians widely report VR as an effective teaching technique, helping to alleviate recall bias.^23,24^ Furthermore, a previous review of clinicians’ attitudes towards NRVR found broadly positive attitudes, with improved time perception, self-awareness, and protocol compliance cited as key benefits for staff.^25^

Despite largely favorable evidence, NRVR has been slow to gain widespread adoption. This may be due to perceived difficulties overcoming legal barriers of recording medical events, logistics of recording emergency situations, and privacy concerns from patients and staff members.^26^ Prior research using NRVR has focused on neonatal outcomes, but guidance about additional challenges or potential pitfalls of NRVR has not yet been comprehensively addressed. Examining participants’ experiences may assist in improving NRVR for both the purpose of debriefing the attending team and for teaching other staff members who weren’t present at the resuscitation.

**Fig. 1:**
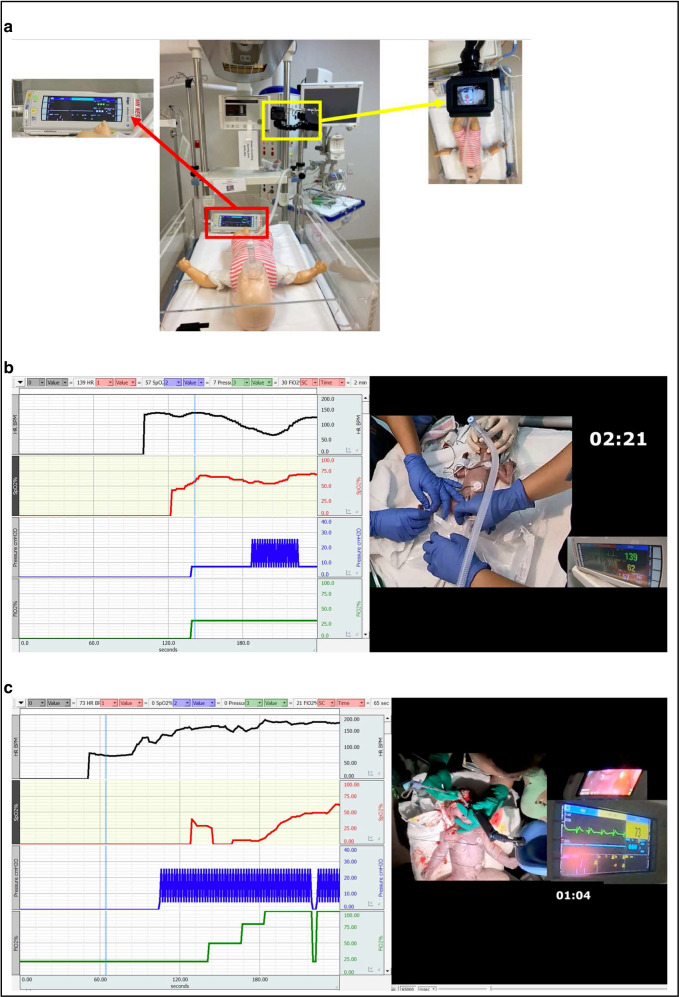
NRVR camera arrangement and NRVR video stills. **a** Resuscitaire with a GoPro camera attached as used to record NRVR videos. **b** NRVR video of an extremely preterm newborn being supported with a facemask with continuous positive airway pressure after delayed cord clamping, alongside paired physiologic data. **c** An infant with congenital diaphragmatic hernia who is being intubated before umbilical cord clamping after a vaginal birth. In this example, the mother’s right foot and the screen from the video laryngoscope are visible. The vertical blue line scrolls in time across the graph and correlates physiologic data with the time from birth. Parental consent was provided for the use of these images.

At Monash Newborn (a tertiary NICU—neonatal intensive care unit—in Melbourne, Australia), NRVR sessions reviewing video recordings (with audio) paired with physiologic data have been ongoing since 2019 (Fig. 1). By qualitatively evaluating clinicians’ experiences, educational programs can be further improved, optimized, and expanded. Therefore, we aimed to evaluate clinicians’ attitudes and experiences of using NRVR.

## Methods

A qualitative approach was selected to examine individual clinicians’ in-depth responses to NRVR, using a social constructivist approach, in which, learning occurs through interaction with the help of others.^27^ The processes of NRVR align with social constructivism, as clinicians watch a resuscitation and are then guided by a facilitator as they observe and reflect on the event.

### Neonatal resuscitation video review procedures

Video recordings are taken of resuscitations of infants born at <32 weeks gestation or otherwise anticipated to require respiratory support at birth (e.g., congenital diaphragmatic hernia). Video recordings (GoPro, USA) of the newborn on the resuscitaire and the monitor (Drager, Infinity M450, Germany) were then edited (DB) (Movavi Video Editor 23, USA) to show a birds-eye view showing the infant with the hands of the clinical team, as well as the monitor screen. To focus viewers’ attention on the team’s actions (versus any individual’s performance), clinicians’ faces are not visible, but their voices and background noise are retained. The neonate’s physiological data (heart rate, peripheral oxygen saturation, inflation pressures, and fraction of inspired oxygen) are recorded by the second into an Excel spreadsheet and then converted to a graph and paired with the video (Fig. 1, Biopac, Acqknowledge, USA).

Videos are selected (DB) for group review either to showcase a particular scenario or to discuss teaching points from the resuscitation (e.g., strengths and areas for future improvement). If a video captures a significant deviation from the standard of care or a potentially distressing event to the clinical team, like a death during resuscitation, this is escalated to the director of the NICU for input prior to being shown.

### Description of NRVR sessions

#### Individual session

Clinicians who have been recorded leading a resuscitation may first review their video alongside a senior doctor (DB). In an individual review session, the recording is watched in full without interruption until physiological stability is reached. Then, the video is restarted and paused frequently to identify moments of clinical importance, facilitate review of the resuscitation, and identify points for future improvement. After individual review, clinicians may decline to show the video in group settings.

#### Group session

Group sessions included 4–10 individuals, which may or may not include the clinicians who attended the birth and are on the video being presented. At the start of each session, all participants sign an agreement to maintain confidentiality regarding the identity and performance of recorded individuals and any discussion during the session. This written agreement includes the Basic Assumption “We believe that everyone seen in this video and participating in video review is intelligent, capable, care about doing their best, and want to improve”.^28^ The facilitator repeats the Basic Assumption verbally prior to starting the session. As with individual reviews, the recording is first shown fully to provide viewers an overview of the resuscitation. After a pause to discuss initial impressions, the video is restarted. The facilitator (DB, a senior doctor experienced in video teaching) pauses the video frequently and leads the discussion. Key focus areas include preparation for resuscitation, communication quality, clinical decision-making, and the physiological response of the newborn. When the video is paused, attendees are invited to discuss what happened and encouraged to ask questions.

During the study period, regular video review teaching sessions (1–3 per month) were held separately for doctors and nurses. This is partly due to scheduling difficulties in aligning protected teaching for nurses and doctors and partly to maintain smaller group sizes. Clinicians in training (e.g., registrars, fellows, junior nurses) are encouraged to attend NRVR sessions as part of their learning. Rotating medical or nursing students are also permitted to attend NRVR sessions.

### Study participants

The study population consisted of doctors and nurses who had attended an NRVR session within the previous 6 months. Potential participants were recruited via email or text containing an explanatory statement about the study. Interested participants returned contact, and an interview was arranged. Interviews were conducted with written consent in a private room or via Zoom.

Interview transcripts were regularly reviewed to ensure clinicians of a range of experience levels were represented in the study participants. In the later part of the study period, a purposive approach was taken to recruitment to ensure that senior clinicians were adequately represented.

### Data collection

All interviews were conducted (by ZW or AK) using a semi-structured interview guide developed by the research team (Appendix 1) and pilot-tested before data collection. Questions included topics such as resuscitation (e.g., “In your opinion, what makes a resuscitation go well?”), and regarding their experiences of NRVR (e.g., “How do you find the experience of reviewing videos in a group?”). Where possible, questions were open-ended to generate discussion without limiting responses.

Before the interview commenced, participants were advised that their responses were confidential, and that identifying data would not be shared beyond the research team. Interviews were audio-recorded, and a verbatim written transcript was produced using transcription software (Otter.ai, USA), with identifying data (e.g., names) removed manually. The audio recordings were reviewed to check nuances were accurately interpreted and transcripts were reviewed for accuracy. Participant characteristics (e.g., job title and experience level) were also recorded. Participants could listen to the interview recording or read the transcript if desired. Repeat interviews were not undertaken.

### Data analysis

Transcripts were analyzed following Braun and Clarke’s six-step model of reflexive thematic data analysis.^29^ Five researchers (ZW, DB, AK, DN, and AB) shared the transcript analysis, and each transcript was examined and coded by at least two researchers. Coding involved identifying ideas or concepts that a researcher finds interesting in a participant’s response.^29^

Following this, multiple meetings were held, during which researchers presented their coding results.^29^ Where there was variation in coding results, these were discussed by all present to reach a consensus. Codes that recurred frequently and were considered important by the research team were identified. During these meetings, multiple theoretical perspectives were considered in interpreting coding results, with Kolb’s Experiential Learning theory used to construct the process of participant learning in NRVR.^30^

Related or similar codes were then revised into themes, which aimed to identify the underscoring ideas in participant responses. These themes were also discussed, reviewed, and refined.^29^ A reflexivity statement detailing each researcher’s role and relevant experience is in Appendix 2.

### Theoretical underpinning

**Fig. 2:**
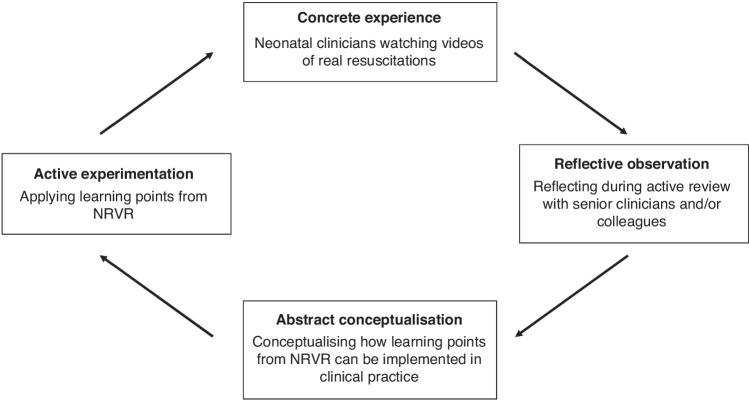
Kolb’s learning cycle adapted to reflect NRVR, adapted from Kolb.^30^

Kolb’s Experiential Learning Theory was used as a theoretical underpinning to understand participant responses to NRVR (Fig. 2). Kolb describes learning as a four-stage cycle, wherein following a “concrete experience” (i.e., participation in NRVR), the learner reflects on their experience (e.g., observing the video, reflecting and engaging in the review), identifies relevant learning points (i.e., abstract conceptualization), and from these earlier stages, puts new skills into practice; “active experimentation”.^30^

## Results

### Participant characteristics

Twenty-two of the 52 eligible participants were enrolled and interviewed, including ten doctors (registrars, fellows, and consultants), 11 nurses (registered nurses and clinical nurse specialists), and one nurse practitioner (Table 1). At Monash Newborn, nurse practitioners work in the same capacity as fellows and are therefore clustered with “doctors” in this study. All participants had attended at least one group NRVR session. Four participating doctors had attended an individual NRVR session. Among nurse participants, none had yet requested to review a video of themselves individually. No eligible clinicians explicitly declined participation; but rather did not respond to recruitment messages. Interviews lasted 9–32 minutes, with a median time of 23 minutes.

**Table 1:** Summary of participant characteristics.

Participant role	Number of participants
Doctors	
Registrars	5
Fellows/nurse practitioners	4
Consultants	2
Nurses	
Registered nurses	5
Clinical nurse specialists	6

Years of experience working in neonatal intensive care	Number of participants
<1	3
1–5	10
5–10	1
10–15	4
15–20	2
>20	2

Gender	Number of participants
Men	3
Women	19

Number of NRVR sessions attended at the time of interviewing	Number of participants
1	11
2	3
3	3
4	1
5+	4

### Themes

**Fig. 3:**
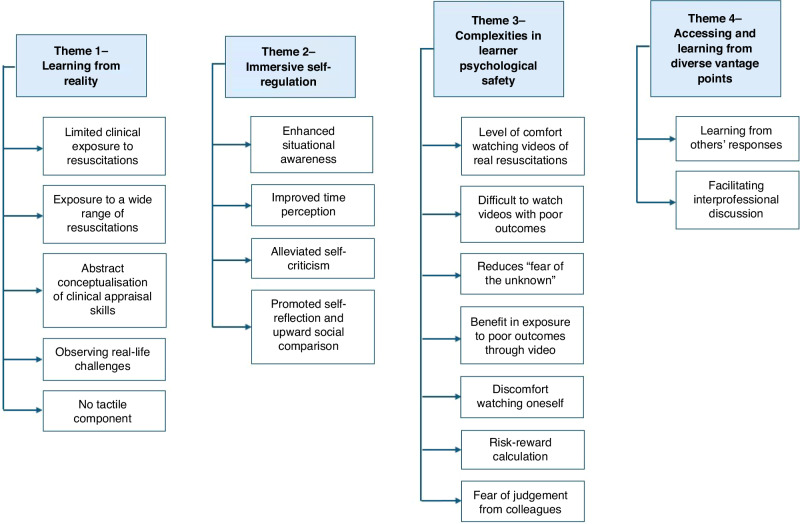
Thematic map of participants’ experiences of NRVR.

All participants reported NRVR as beneficial for learning and indicated a willingness to attend future sessions. Four key themes were identified: 1—Learning from reality, 2—Immersive self-regulation, 3—Complexities in learner psychological safety, and 4—Accessing and learning from diverse vantage points (Fig. 3). Key quotes for all themes are shown in Table 2.

**Table 2:** Themes and subthemes with illustrative quotes.

Theme 1 — Learning from reality	
Limited clinical exposure to resuscitations	“But the reality is that with, what, 20 staff members or more that we have, the actual physical numbers that each staff member, particularly the registrars, are going to go to, is it enough for you to say come out the other end of a six-month term and say I’m good at this?” - Doctor 2 (Fellow) “When we teach students, we might only get the very stable CPAP deliveries and then only when they start attending on their own do they get the unstable intubated patient.” - Nurse 10 (Clinical nurse specialist)
Exposure to a wide range of resuscitations	“I mean, like, the more different experiences, the different scenarios that you see or exposed to, would help you be more prepared to go to a delivery.” - Nurse 9 (Clinical nurse specialist) “But I think I think it would help me feel more prepared, just to have sort of seen what, like, to expect, and have a bit of a framework on what would happen. So, like, for the CDH baby, for example, knowing that they would be intubated immediately. And that’s helpful into seeing what it was like and how they managed it.” - Doctor 8 (Registrar) “But by using this [video review], you’re giving them [junior nurses] the exposure and giving them the education without putting them in a situation that might break them.” - Nurse 1 (Clinical nurse specialist)
Abstract conceptualization of clinical appraisal skills	“You see that baby on the screen go from blue to pink, you see what’s worked, you see how that team has interacted and responded and what’s worked well, and what hasn’t. It’s just much more, I think, real life and relatable to a lot of people.” - Nurse 1 (Clinical nurse specialist) “I think watching the actual video of the baby in real life who started to turn pink when you started giving IPPV and going up on the oxygen, and then you can see what the [oxygen] sats are doing. So, it’s a lot more helpful watching.” - Nurse 2 (Clinical nurse specialist)
Observing real-life challenges	“You can see how tricky it is to wipe the baby and get all of the fluid off to make sure that the sticky chest leads stick, like, you know, it’s just those little practical things that you just can’t do when you’ve got a dummy in front of you.” - Nurse 9 (Clinical nurse specialist) “There’s difficulty in finding certain equipment or delaying a procedure or, you know, things don’t quite run to the algorithm, not through anyone’s fault, but just because that’s how real life works. And so, sitting with that it helps you develop that comfort with uncertainty a bit better.” - Doctor 5 (Registrar)
No tactile component	“To really solidify that learning you need to be in that situation yourself or have a chance to practice those skills in like a safe environment. So, I think that it’s [NRVR] a really good tool. That could also be an even better tool if it was an adjunct to more sim-based learning as well.” - Doctor 1 (Registrar) “There’s probably ways that you can, sort of, like watch a resus video, and then sort of, you know, the next session that week or the next session next week, you can have, like, a more practical session, and sort of, like, maybe practice those principles that you talked about.” - Doctor 3 (Registrar)

Theme 2 — Immersive self-regulation	
Enhanced situational awareness	“It’s really easy to hone in on, you know, providing IPPV to a baby and not thinking about the other things like is the mask the right size? Am I using the right pressures? Do I need to increase oxygen? Should we change the position? When you’re in that moment, it’s really, really easy to get tunnel vision.” - Nurse 1 (Clinical nurse specialist) “You can get a lot out of seeing how you do things from the third person perspective. Yes, how you do things, how others do things. And watching them slower, or, or over and over again. Because you always find new details that you can comment on, be they positive or negative” - Doctor 11 (Neonatologist)
Improved time perception	“I think it’s good to, like, dissect, like you can go a lot slower. And you know, when [facilitator] goes through, pauses like, ‘What was happening here?’ and then you’re like, Wait, there’s a lot more happening that we can actually process, that you may not have been able to do in real-time, or actually get a sense of, like, time. Sometimes time moves very strangely in a resuscitation.” - Doctor 1 (Registrar) “Probably the time, like, it feels like it’s a long time. But when you watch it back are like, actually, that was quite quick” - Doctor 7 (Fellow)
Alleviated self-criticism	“It was a positive thing to have gone back and reviewed it and be able to, like, readjust. ’Oh, that [resuscitation] was dreadful, like, that was the most terrible worst thing that could possibly have happened, that was awful.’ But it actually wasn’t, and to try and, like, rationalise it and be a bit more objective and made it much more objective. So, when it’s just your personal memory, it’s a very subjective review.” - Doctor 7 (Fellow) “Sometimes I think I might get a bit stuck in my head thinking about things. So, to kind of actually watch in real time, how you do perform, I might realise I do a little bit better than I think I do.” - Nurse 6 (Registered nurse)
Promoted self-reflection and upward social comparison	“I think especially like, in a nursing role, as well, like, you can see how different nurses communicate with the team. And like, you can compare that to yourself and like, take kind of, yeah, take it as, like an educational point to apply to your practice.” - Nurse 4 (Clinical nurse specialist) “I think watching other how other people do things, helps you decide how you want to do things as well and, and what makes sense to you, and what doesn’t necessarily make sense to you.” - Doctor 3 (Registrar)

Theme 3 — Complexities in learner psychological safety	
Level of comfort watching videos of real resuscitations	“It’s confronting but obviously less confronting is than it is when you’re actually there.” - Nurse 4 (Clinical nurse specialist) “I can kind of look at it [video review] with, you know, less emotional view, more, like, a learning view, rather, whereas there will be some people that will be quite, find it quite confronting.” - Nurse 8 (Clinical nurse specialist)
Difficult to watch videos with poor outcomes	“If there was a massive bleed or something, or you could see that stuff was going really wrong. And people are trying their best, but they can’t do it, I think it would actually be more confronting on the video, rather than in real life” - Doctor 1 (Registrar) “I think if there was a terrible outcome, I think that’d be difficult.” - Doctor 4 (Fellow)
Reduces “fear of the unknown”	“And, if I had been played that video back before I’d done anything, I would have been a lot less scared to go to deliveries.” - Nurse 3 (Registered nurse) “I think it would be, like, more of a gentle introduction to deliveries. And then, like, once you’ve got an idea of how a resuscitation should work, then you can sort of add in all those extra elements that you need to learn and remember as well.” - Nurse 6 (Registered nurse)
Benefit in exposure to poor outcomes through video	“Like, it’s all good seeing everything go well and smoothly. But it also would be beneficial to have, you know, a video of a resus that maybe like required some chest compressions and umbi lines. Because I did get put into that situation once where I went down to take some equipment to ED and ended up being part of a resus doing drugs that was, you know, chest compressions and umbi lines and everything. And sadly, that baby didn’t make it. But I think it would have been good to have been a bit more prepared for what that situation looks like.” - Nurse 6 (Registered nurse) “The baby [in the video] was extremely pale and really flat… And I think watching that was kind of like a, this could be me in a peripheral hospital receiving a really unwell neonate.” - Doctor 8 (Registrar)
Watching videos of oneself can be uncomfortable	“I think it would be quite like anything like rewatching anything that you’ve done is always mild to moderately painful.” - Doctor 1 (Registrar) “Oh, it’s always awkward to hear your own voice. And see your own face if that happens. So awkward, but I hope that my performance of what I did for the baby would be useful in learning something” - Nurse 10 (Clinical nurse specialist)
Risk reward calculation — benefits of NRVR supersede discomfort watching self	“I hate it [watching self on video]. But I’ve become more, trained myself to be more comfortable with it. Because we’ve had the opportunity to have video review before. And so, I mentally know that it’s good for me. And it will improve what I do. And so yeah, so I think it’s just a matter of, like, forcing yourself to do it. And then it’s actually not as bad as you think.” - Doctor 7 (Fellow) “Maybe afterwards, it’s a bit embarrassing to listen to your own voice. But it’s good to learn. And it’s good to see how you interacted with other people as well. I’d be up for it, I wouldn’t mind at all.” - Doctor 9 (Registrar)
Fear of judgment from colleagues	“I think there is a bit of a perception of there might be some criticalness of other team members. And it makes people hesitant to do it because of the fear of critique. So, I’m not worried about it. My impression is that some people worry about that stuff. That may, that might, be a block for some people.” - Doctor 2 (Fellow) “I feel like it would make me a bit more nervous. Like, oh, everyone’s gonna be seeing this and knowing that it’s me.” - Nurse 11 (Registered nurse)

Theme 4 — Accessing and learning from diverse vantage points	
Learning from others’ responses to NRVR	“And I think it exposes us to discuss these things, you know, you learn a lot by having a multi-perspective discussion because we see different things. And so, there’s always things that I didn’t notice that other people are focusing on.” - Doctor 4 (Fellow) “I think it’s good because you can also learn from your peers. They can say, you know, in that situation, I probably would have done X, Y, or Z if they’ve had prior experience.” - Nurse 9 (Clinical Nurse Specialist)
Facilitating interprofessional discussion	“I liked it to have a multi-disciplinary group rather than just nurses teaching nurses. Good to have a doctor in there… it’s good if we learn to do our resuses as a team rather than you learn this and we learn that and then you’re supposed to work together when we get to it, I think as a teaching team, so then if you’re reviewing, we need to review it as a group, as a team as well.” - Nurse 8 (Clinical nurse specialist) “We [nurses] probably look at certain things differently, compared to how doctors look at it, so good to hear ideas from both sides.” - Nurse 2 (Clinical nurse specialist)

### Theme 1 — Learning from reality

NRVR utilises videos of clinical situations, making this highly relatable to clinical practice. This relatability to clinical practice was a key benefit of NRVR, as it made learning more transferrable to real resuscitations.

#### Exposure to a wide range of resuscitations

The number of clinicians rostered to attend neonatal resuscitations is larger than the number of resuscitations that occur. Several doctors and nurses noted that their clinical exposure was insufficient to make them feel competent. As NRVR allows clinicians exposure to a range of resuscitations that wouldn’t otherwise have been possible, many clinicians reported this helped them feel more prepared for a diverse range of resuscitations.

#### Enabled abstract conceptualization of their clinical appraisal skills

Many clinicians noted that NRVR sessions allowed them to observe how an infant’s condition changes in response to interventions, which allowed them to use their clinical appraisal skills vicariously. Secondly, participants reported that NRVR exposed them to the “messiness” of real resuscitations. Many participants spontaneously compared NRVR to simulation teaching in this regard, noting that this allowed them to practice their troubleshooting skills. However, NRVR sessions lack hands-on practice of clinical skills, which several participants noted would be beneficial.

### Theme 2 — Immersive self-regulation

NRVR presented participants with a model that enabled them to access their memories of previous resuscitations they had attended. Doing so resulted in enhanced situational awareness, improved time perception, and alleviated some of their self-criticisms, and promoted self-reflection and upward social comparison.

#### Enhanced situational awareness and improved time perception

Many participants noted that the high stress of resuscitations caused them to sometimes experience “tunnel vision,” wherein, through focusing on their task, they lost situational awareness. This, in turn, led them to have a limited understanding of the broader resuscitation. Furthermore, participants also reported their recall of resuscitations that they had attended was often limited, especially their perception of time. Video review and the associated discussions helped them to gain perspective and calibrate their perception of time.

#### Alleviated self-criticism

Participants who reviewed videos of themselves reported that NRVR provided a more objective assessment of their management, reducing their recall bias. NRVR enabled them to recalibrate and realign their self-judgment to reality, alleviating lingering self-criticism.

#### Promoted self-reflection and upward social comparison

Many participants reported that watching videos of others also prompted them to reflect on their behaviors. Several described how the videos allowed them to compare themselves to their recorded colleagues in the same role, an opportunity rarely afforded in daily practice. They then reported using their colleague’s performances as models of behavior to be emulated.

### Theme 3 — Complexities in learner psychological safety

Partaking in NRVR is not without challenges. Multiple elements may cause discomfort for participants, such as the content of the videos—neonates receiving medical interventions, watching recordings of themselves, or a fear of being judged when recordings are shown to others.

#### Level of comfort with watching videos of real resuscitations

All participants reported being generally comfortable watching videos of resuscitations. This was consistent across professions (i.e., doctors and nurses), seniority levels, and participation in group and individual NRVR sessions. Most participants were concerned that NRVR could be confronting to others, particularly those who had had limited exposure to resuscitations. However, many times, participants noted that they found that seeing an unstable infant on video was no more confronting than their clinical practice.

Some participants also noted that it would be difficult to predict their response if they were shown a video of a resuscitation with a poor outcome and endorsed that they did not want to see those videos. However, other participants felt differently—reporting that being exposed to a poor clinical outcome through video would be less confronting than real life and could have helped reduce their fear of the unknown.

#### Risk-reward calculation — benefits of NRVR supersede discomfort

Participants who had not reviewed videos of themselves generally noted that they would feel some self-consciousness watching videos of themselves. However, participants widely reported the educational benefits of NRVR superseded this discomfort.

Participants who had been able to watch videos of themselves reported similar sentiments, noting that they were willing to experience the discomfort of watching themselves for the educational benefit, and generally reported less anxiety around the process of watching themselves compared to participants who had not yet watched themselves. While most participants felt NRVR group sessions were supportive, some feared being judged by their colleagues, with some junior clinicians, particularly nursing staff, expressing trepidation around being recorded or contributing to group discussion. A few participants noted that this may be a barrier towards broader acceptance of NRVR.

### Theme 4 — Accessing and learning from diverse vantage points

Both doctors and nurses relayed that it was beneficial to review videos with colleagues—as this helped expose learners to new perspectives and allow knowledge to be shared. Several nurses interviewed also reported that, as NRVR sessions are facilitated by a doctor, this promoted interprofessional conversation, and allowed them to be better exposed to doctors’ views and roles in resuscitation. However, some nurses also suggested the addition of a nurse facilitator as a potential improvement — to improve the quality of feedback on nursing-led tasks.

## Discussion

The purpose of this study was to capture clinician’s experiences of NRVR. The results demonstrated that participants found NRVR sessions highly relevant and applicable to their clinical practice, and watching videos of their colleagues or themselves helped promote self-reflection. There can be psychological safety challenges associated with participating in NRVR. Participants reported that partaking in NRVR can lead to being self-conscious about watching themselves; however, they reported that the educational benefits of NRVR superseded this concern. This was consistent between doctors and nurses, participants of different seniority levels, participants who had attended group and individual NRVR sessions, and participants who had attended one NRVR session compared to those who had attended multiple.

Furthermore, NRVR is an effective and feasible technique for increasing clinician exposure to low-frequency events, such as neonatal resuscitation. Through the perspective of Kolb’s Experiential Learning theory, videos of real neonatal resuscitations can be viewed as a “concrete experience.” Through discussion of these videos in NRVR sessions, clinicians can observe and reflect via active review. After NRVR sessions, participants conceptualize how teaching points can be implemented in the future and finally implement these changes to their clinical practice, representing the final “active experimentation” stage.

The results of this study are broadly consistent with previous investigations of clinicians’ attitudes toward video review of neonatal and other emergencies, finding situational awareness, improved time perception, and clinical exposure to be key benefits to NRVR.^23,25,31^ By focusing on participants’ experiences of NRVR, this study adds new knowledge to this topic; including highlighting the crucial need to attend to participants’ psychological safety so that they make the most of their participation. The results of this study add positive clinician attitudes to the other published benefits of NRVR—such as increasing guideline compliance in resuscitation and allowing for areas of improvement to be identified.^17,22^

### Strengths

A key strength of this study was that data analysis was shared between five researchers from different disciplines. This collaboration helped mitigate the impacts of individual researcher bias. Additionally, as an even number of doctors and nurses were interviewed, the results of this study are not particularly limited to a certain profession. As doctors and nurses typically have different roles in resuscitations, it was essential that both perspectives be examined adequately. Furthermore, a purposive approach to recruiting ensured the interviewees in our study had a wide range of experience levels, and a range of perspectives were examined.

Through the data analysis process, several suggested improvements of the NRVR program were identified—such as running multidisciplinary sessions with both medical and nursing facilitators and incorporating hands-on practical skill elements. These suggestions will guide future changes to the Monash NRVR program.

### Limitations

There were some disparities in participant characteristics that may impacted the findings. There was a gender disparity, as only three doctors interviewed were men (27%), and no nurses who identified as men were interviewed. While this ratio is relatively consistent with the workforce gender disparity (as of 2017, 11% of nurses in Australia were men, and as of 2022, 46% of medical trainee doctors in Australia were men), this may have impacted the data.^32,33^ As no nurses had taken part in individual NRVR sessions, we were not able to evaluate if experiences of individual review varied between doctors and nurses. Furthermore, as group NRVR sessions were run separately for nurses and doctors, we were not able to evaluate the impact of interdisciplinary discussion.

It is possible that clinicians with more negative attitudes towards NRVR chose not to participate. As no eligible clinicians explicitly declined participation, data was not collected regarding reasons for declining participation.

As the consultant who organizes and facilitates NRVR sessions was a member of the research team, participants may have felt pressured to report positive attitudes. While participants were encouraged to share negative comments during the interviews and the organizing consultant was not involved in the interview process, this may have affected the data collected.

Furthermore, as NRVR has been running as an educational program at Monash Newborn since 2019, participants may have become accustomed to it. It is possible that, if this study had been conducted at the time NRVR was introduced, participants would have reported more anxiety and trepidation around its use.

## Conclusion

This study demonstrated that neonatal doctors and nurses found NRVR to be valuable and educational—particularly in increasing situational awareness, clinical exposure, and providing discussion opportunities. Importantly, participants also reported that they generally did not find NRVR confronting. Those facilitating NRVR should actively monitor participants for discomfort and promote constructive group conversation to maximize educational benefits.

Ensuring that all healthcare workers receive frequent refresher training is essential in improving performance in neonatal resuscitation.^6,8^ NRVR shows potential as standard initial training of new staff and as ongoing training for experienced clinicians. Further study is warranted to determine if NRVR improves clinical outcomes.

## Disclaimer

The views expressed herein are those of the author(s) and do not necessarily reflect the official policy or position of the Defense Health Agency, Uniformed Services University of the Health Sciences, the Department of Defense, or any agencies under the U.S. Government.

## Supplementary information

Supplementary Materials

## Data Availability

The data (interview transcripts) generated during and analyzed during the current study are not publicly available to protect participant confidentiality but are available from the corresponding author on reasonable request.
