# Supplementary material for: Impact of the Neonatal Resuscitation Video Review program for neonatal staff: a qualitative analysis
Source: Pediatr Res. 2024 Oct 4;97(7):2272–81. doi: 10.1038/s41390-024-03602-9 (PMC12279539; doi:10.1038/s41390-024-03602-9)
Supplement: Supplementary file 1 — Supplementary Materials [file 41390_2024_3602_MOESM1_ESM.docx]

**Appendix 1 – Interview Guide**

**Section 1 – Demographic information**

- What is your occupation at Monash Newborn?
- How long have you worked in neonatal intensive care?
- Have you been able to attend neonatal resuscitations before? If so, roughly how many neonatal resuscitations have you attended?

**Section 2 – Quality of resuscitations**

- In your experience, what makes a resuscitation go well?
- In your experience, what makes a resuscitation go poorly?
- How would you describe the resuscitations that you have been involved in at Monash Newborn?
- How would you describe the teamwork and communication in the resuscitations that you have been involved in?
- How do you feel before you go to a neonatal resuscitation?

**Section 3 – Experience of NRVR**

- How many NRVR sessions have you attended?
- How have you found the NRVR sessions that you’ve attended?
- How do you find the experience of watching videos in a group?
- Are there any changes to your clinical practice you are thinking of making after attending NRVR sessions? If so, what are they?
- Is there anything that you did not like about the NRVR session?
- Is there anything that you would change about how NRVR sessions are run?
- Do you have any concerns about the use of NRVR at Monash Newborn?
- How would you feel about watching a video of yourself in a resuscitation?
- How would you feel about others watching a video of you in a resuscitation?

**If the participant has reviewed videos of themselves**

- How did you find the experience of watching a video of yourself?
- (If applicable) How did you find the experience of your video being shown to a group?

**Appendix 2 – Reflexivity Statement**

In qualitative analysis, the experiences of the researchers involved may affect the way that the data is collected and understood.^29^

All interviews were conducted by ZW (a medical student with no previous experience in qualitative research). AK (a consultant obstetrician-gynaecologist experienced in qualitative data collection) was present to supervise the first 3 interviews to ensure appropriate quality and depth of the interviews. Following this, ZW conducted all further interviews alone. Neither ZW or AK were involved in NRVR teaching, or professionally involved at Monash Newborn. No other researchers or individuals were present during interviews.

Interview analysis was shared between ZW, AK, DB, DN, and AB. DB is a consultant neonatologist who facilitates NRVR sessions and does not have prior experience in qualitative research. He was not involved in recruiting participants or conducting interviews. He is professionally connected to all participants.

DN is a professor of simulation education in healthcare, who has significant experience in qualitative research methods and evaluation of clinical education programs. She has no professional or personal connections to the participants and was not involved in the interview process.

AB has a PhD in educational psychology and is professionally involved in teaching graduate research methods courses. She has significant experience in qualitative research. She has no professional or personal connections to the participants and was not involved in the interview process.
